# Interrogating the World Bank’s role in global health knowledge production, governance, and finance

**DOI:** 10.1186/s12992-021-00761-w

**Published:** 2021-09-19

**Authors:** Marlee Tichenor, Janelle Winters, Katerini T. Storeng, Jesse Bump, Jean-Paul Gaudillière, Martin Gorsky, Mark Hellowell, Patrick Kadama, Katherine Kenny, Yusra Ribhi Shawar, Francisco Songane, Alexis Walker, Ryan Whitacre, Sumegha Asthana, Genevie Fernandes, Felix Stein, Devi Sridhar

**Affiliations:** 1 Department of Anthropology, Durham University, Dawson Building South Road, Durham, DH1 3LE UK; 2 Global Health Studies, Department of History, University of Iowa, 280 Schaeffer Hall, Iowa, 52242 USA; 3 Center for Development and Environment, University of Oslo, Norway, Postboks 1116, Blindern, 0317 Oslo, Norway; 4 Department of Global Health and Population, Harvard University T H Chan School of Public Health, 665 Huntington Avenue, Building 1, Room 1205, Boston, MA 02115 USA; 5 Centre de recherche médecine, science, santé et société (CERMES3), Ecole des Hautes Etudes en Sciences Sociales, 7, rue Guy Môquet, 8 - 94801 Villejuif Cedex, BP France; 6 Centre for History in Public Health, London School of Hygiene and Tropical Medicine, UK, Room S12, LSHTM, 15-17 Tavistock Place, London, WC1H 9SH UK; 7 Global Health Policy Unit, Social Policy, University of Edinburgh, Chrystal Macmillan Building, 15A George Square, Edinburgh, EH8 9LD UK; 8 African Center for Global Health and Social Formation, Plot 13 B Acacia Avenue, Kololo, P.O. Box 9974, Kampala, Uganda; 9 Department of Sociology and Social Policy, University of Sydney, Australia, A02 - Social Sciences Building, Camperdown, NSW 2006 Australia; 10 Bloomberg School of Public Health and Paul H. Nitze School of Advanced International Studies, Johns Hopkins University, 615 N. Wolfe Street Room E8132, Baltimore, MD 21205 USA; 11 Africa Public Health Foundation, 5th Floor, The Atrium Kilimani, Nairobi, Kenya; 12 Columbia University Irving Medical Center, 630 W. 168th St., New York, NY 10032 USA; 13 Global Health Centre, Graduate Institute of International and Development Studies, Case postale 1672, 1211 Genève 1, Switzerland; 14 Center of Social Medicine and Community Health, Jawaharlal Nehru University, New Mehrauli Road, New Delhi, 110067 India; 15 Usher Institute, University of Edinburgh, Old Medical School, Teviot Place, Edinburgh, EH8 9AG UK; 16 Centre for Development and the Environment, University of Oslo, Postboks 1116 Blindern, 0317 Oslo, Norway

**Keywords:** World Bank, International health policy, Power, Epistemology, Governance, Finance, Global health

## Abstract

**Background:**

In the nearly half century since it began lending for population projects, the World Bank has become one of the largest financiers of global health projects and programs, a powerful voice in shaping health agendas in global governance spaces, and a mass producer of evidentiary knowledge for its preferred global health interventions. How can social scientists interrogate the role of the World Bank in shaping ‘global health’ in the current era?

**Main body:**

As a group of historians, social scientists, and public health officials with experience studying the effects of the institution’s investment in health, we identify three challenges to this research. First, a future research agenda requires recognizing that the Bank is not a monolith, but rather has distinct inter-organizational groups that have shaped investment and discourse in complicated, and sometimes contradictory, ways. Second, we must consider how its influence on health policy and investment has changed significantly over time. Third, we must analyze its modes of engagement with other institutions within the global health landscape, and with the private sector. The unique relationships between Bank entities and countries that shape health policy, and the Bank’s position as a center of research, permit it to have a formative influence on health economics as applied to international development. Addressing these challenges, we propose a future research agenda for the Bank’s influence on global health through three overlapping objects of and domains for study: knowledge-based (shaping health policy knowledge), governance-based (shaping health governance), and finance-based (shaping health financing). We provide a review of case studies in each of these categories to inform this research agenda.

**Conclusions:**

As the COVID-19 pandemic continues to rage, and as state and non-state actors work to build more inclusive and robust health systems around the world, it is more important than ever to consider how to best document and analyze the impacts of Bank’s financial and technical investments in the Global South.

**Supplementary Information:**

The online version contains supplementary material available at 10.1186/s12992-021-00761-w.

## Background

Within six years of establishing a division for direct lending for health in 1979, the World Bank^1^ became the largest financial investor in health to countries in the Global South [42]. Since then, the Bank has been consistently among the most influential actors in global health [11, 56]. Alongside its direct lending, it has positioned itself as a self-described “Knowledge Bank” [43, 80], producing expertise on health policy and financing, and has taken on new roles within global health partnerships and trust funds. Because of its considerable sway in global health and vast financial resources and networks, the World Bank’s influence on health sector priorities and resource mobilization has been and should continue to be an object of scrutiny. As a development bank whose main modalities are government loans and grants, and whose twin mandate is to end extreme poverty and support shared prosperity, the Bank has a unique perspective in the global health landscape. Further, the institution’s global health influence sits within its considerable financial influence on international development as a whole [22]. How, then, can social scientists and historians interrogate the World Bank’s varied influence on “global health” in the current era? How can we improve the relevance of research findings, for holding the Bank to account, recognizing its successes and the contexts in which they are actually reproducible, and assuring that the Bank’s perspectives on health policy are balanced by perspectives from the countries in which it works? The COVID-19 pandemic has shown how crucial strong, flexible, and inclusive health systems are for guaranteeing the well-being of the world’s population, and it has also made it clear how the conditions by which health systems are constructed are fundamental to how they perform under such pressure. It has become all the more important to consider how social science research can better document the World Bank’s influence on health systems in the Global South, and the ways it exerts this influence.

How to best study the World Bank’s influence and power in global health was the topic of a symposium that brought together experts on the topic at the Brocher Foundation in January 2019, under the auspices of Devi Sridhar’s Wellcome Trust-funded project, *The Economic Gaze: The World Bank’s Influence in Global Public Health*. The project’s research (conducted by Fernandes, Sridhar, Stein, Tichenor, and Winters) focused on the influence of the Bank’s “traditional” arms of development aid: the International Development Association (IDA) and the International Bank for Reconstruction and Development (IBRD). From 2016 through 2019, among other research activities, the project team performed extensive literature reviews of the Bank’s past work in global health, which grappled with its widespread “influence” in global health governance. These included the Bank’s own narratives, which largely described its influence in terms of financial allocations to various health sector themes and dissemination of key health sector policy papers (e.g., [14]). They also included narratives produced by the Independent Evaluation Group (IEG), which are unique because they are both internal and external: the group is constituted of independent evaluators of the Bank’s work but it reports to the Executive Board and is shaped by the World Bank Group Strategy. From this internal perspective, the Bank emphasized poverty alleviation and population control in the 1970s, increasing focus on primary health care and “health reform” from around 1987–1996, and the sector-wide approach for health system strengthening from 1997 to the mid-2000s. These internal narratives also include flagship reports the Bank has disseminated about its own vision of health knowledge, governance, and financing. This includes its seminal 1993 World Development Report, “Investing in Health,” which Dean Jamison – one of its architects – described as a “centre left report from a centre right institution” ([36]:1871; [81]). As such, the report has been criticized from various technical and political perspectives [50], including that it had the potential to infuse all corners of health policy and governance with the logics of economics (e.g. [38, 71]) and that public investment in education would be better than health for stimulating economic growth [77].

Political science, health policy, anthropology, and history studies add nuance to this health sector theme-based narrative of the Bank’s influence, particularly by relating it to other players in the global health governance landscape. These studies often challenge the positivism of internal Bank narratives, such as by theoretically grounding the concept of “health reform” in neoliberalism (e.g., [37, 47]). In the first external, comprehensive study of the Bank’s work in the health sector since the 1960s, for instance, Kamran Abbasi explained that the Bank eclipsed the World Health Organization (WHO) in influence in the 1980s due to its financial prowess, and he pointed to controversial themes raised by its “critics,” including the Bank’s structural adjustment and user fee policies in the late 1980s, and its embrace of private health care provision and disability adjusted life years (DALYs) during the 1990s [1, 2]. Sophie Harman has further argued that the Bank’s market-driven health ideologies have become so strategically ingrained in global health governance that the Bank “no longer needs to use large loans with stringent conditionalities to influence global health” ([25]: 242), and Devi Sridhar has described how human capital theories contributed to extending the reach of the Bank’s influential “economic gaze” on global health [62, 63].

The Bank’s influence on health policy knowledge production, governance, and financing has been tackled directly or indirectly by a growing number of case studies, most of which have never been compiled or collectively analyzed. These studies variously describe the Bank’s influence in terms of its power to guide priority-setting through its creation and dissemination of efficiency-grounded metrics; its power to leverage financial assets and a financial management approach to promote specific types of health investments; and its power to guide decision-making, relative to other global health institutions and nations. Based on research conducted by the *Economic Gaze* project team and consensus-making discussions during and after the symposium, we propose three research ‘domains’ that may help to better capture the World Bank Group’s (WBG’s) nuanced influence in the global health realm: (1) knowledge-based (how it is produced, who produces it, and what impact it has), (2) governance-based (who is involved in producing health policy and promoting it in spaces of governance), and (3) finance-based (how financing mechanisms are developed and implemented).

Our proposed domains are best viewed heuristically as ‘lenses’ for analysis. Rather than provide a systematic review of all case studies of the WBG’s work in health, this review article focuses on these three domains and how they may inform a future research agenda on the WBG’s entangled forms of power that have contributed to its ability to wield unique influence in global health. As expanded upon in Additional file 1: Table 1, each domain can be conceptualized from two vantage points: the object of study (knowledge, governance, and financing) and the types of power that it best unpacks (discursive, expert, network, institutional, and economic). Our conceptualization of the institution’s influence and power in the global health sphere is informed by Suerie Moon’s [44] expanded typology of power in the global governance space: physical, economic, structural, institutional, moral, discursive, expert, and network. The domains are best viewed *not* as three discrete typologies of influence, but instead as three overlapping ways to more comprehensively approach the Bank’s influence and explore the role of power in the Bank’s work in global health, including its power to shape the language in which research on itself is conducted.

In the main text of this review, we first outline the three intrinsic challenges of studying the World Bank, and then put forward these three major research domains that would promote a more equitable and locally-relevant research agenda on the World Bank Group’s influence in global health. It is important to note that our research agenda was elaborated largely before the COVID-19 pandemic, and particular attention to the ways that the pandemic has reconfigured and will continue to refigure global health governance has not been incorporated into our review. However, considering the sheer amount of funding and technical support that the WBG provides for supporting health, it remains imperative that social scientists and historians strengthen existing research on the organization by building a robust and heterogeneous body of case studies of the WBG’s investments in global health, particularly from those most impacted by these investments.

## Methods

This review of case studies is based on two methods of interrogating the World Bank Group’s influence on global health: document analysis conducted throughout the course of the *Economic Gaze* project and discussions at and after a symposium in January 2019. As described above, document analysis was conducted by the project members (Fernandes, Sridhar, Stein, Tichenor, and Winters) between 2016 and 2019 to identify key case studies and important themes. The symposium was hosted by the Brocher Foundation near Geneva, Switzerland and brought together an international network of historians, anthropologists, economists, political scientists, public health scholars, and representatives of health ministries, all with experience studying or working with the Bank. Scholars and practitioners were selected for participation based on geographic representation and thematic contribution to World Bank and international finance institution analyses, in order to provide a breadth of disciplinary lenses and of institutional perspectives on the many points of entry the WBG uses to shape health knowledge, governance, and financing. This selection process was conducted in two ways. First, the article’s first two authors created a list of potential participants through the review of literature on the World Bank, intentionally seeking out geographic and disciplinary breadth in approaches to analyzing the Bank. Second, they left five of the twenty planned invitations open to early career researchers, whose participation would be fully funded, and widely advertised the application in order to include researchers who were harder to find through the review of literature, particularly those from the Global South. The initial intention of the symposium, titled “Disrupting Global Health Narratives: Alternative Perspectives on the World Bank’s Influence on Global Health,” was to present examples of past case studies from all symposium participants’ research, and to weave them into a narrative of the Bank’s influence on global health. This “counter-narrative” would challenge what we perceived as a relatively “neat” Bank timeline of its own role in the global health sphere, providing a “history from below.”

Through our presentations and discussions at the symposium, and our discussions in the months after, it became clear that, not only is the WBG’s own narrative of its role in global health anything but neat, but some of the areas of anticipated contention (e.g., the generally negative effects of user fees and structural adjustment, which is described further below) had been explored through strong case studies. Yet, others – particularly those related to private sector investment and analysis of power dynamics in the setting of the Bank’s country plans and strategies – were starkly lacking. Furthermore, many of the existing case studies seemed to be operating in their own silos and with different sets of assumptions: many did not cite relevant publications from outside of their academic areas, and the boundaries of what was considered health at the Bank (e.g., nutrition, the traditional organizational arms of the IBRD and the IDA, or the WBG) were often unclear. The review presented here is the result of collective work of gathering case studies, discussing relevant themes, and identifying crucial gaps to be filled with future research. The framework for structuring this review – based on Suerie Moon’s [44] typology of power – was chosen based on our months of discussion during and after the symposium, and with the very helpful guidance of our anonymous reviewers.

## Three challenges to studying the World Bank’s role on Global Health

In order to understand our proposed three domains for future research, we must first outline three central challenges to studying the WBG’s role in global health. These challenges were first identified during discussions at the symposium, and include: the WBG’s internal complexity, its shifting approaches to health over the course of more than 40 years of health investment, and its entanglement with a growing number of actors in the global health space.

The first challenge requires recognizing that the World Bank is not a monolith, and its health portfolio extends beyond its Health, Nutrition, and Population (HNP) division, such that it is essential to carefully consider how researchers are defining the World Bank and whether their definition is appropriate. The so-called World Bank Group (WBG) is made up of different entities with distinct mandates that place health within a constellation of other development objectives. For this reason as well as the fact that, as a development bank and mentioned above, its main mode of intervention is a loan to a government, the WBG’s approach to health development is distinct from that of the United Nation’s specialized agency for health, the World Health Organization (WHO). The WBG entities – especially the IDA, the IBRD, the International Finance Corporation (IFC), and the Multilateral Investment Guarantee Agency (MIGA) – each include substantial health programs and systems financing in their portfolios. Focusing exclusively on the IBRD and the IDA risks misunderstanding the WBG’s relationship with the private sector, which it has promoted in various forms over the last four decades (particularly through the IFC), as a means for, in its own words, enhancing health equity. Furthermore, alongside the Bank’s (traditional) core HNP sector lending and engagement with the private sector, it is also important to consider its large and often fragmented trust fund infrastructure, as well as the WBG’s projects with health objectives or impacts in allied sectors, such as food security and post-conflict reconstruction.

Second, the WBG’s influence on health policy and investment has changed over time, and the impacts of these policies and investments are often nuanced. In the 1980s and early 1990s, the WBG helped shape the health landscapes in many countries that received funding from the financial institution, as it advocated for private sector funding, the introduction of user fees [6], and other forms of health system self-financing (Akin et al. 1987). Many scholars have argued that the public expenditure reduction requirements in the WBG’s and the International Monetary Fund’s Structural Adjustment Programs were factors that led to many beneficiary countries’ smaller national health budgets [45, 52]. While this has been widely depicted as an embrace of neoliberalism and a driver of reduced national health investments [67], there is also evidence that per capita public health spending in some countries under adjustment either decreased only marginally or actually increased since 2000 [49]. Further complicating the historiography of the Bank’s reform policies, former Bank President Jim Yong Kim signaled a reversal in the Bank’s position on user fees in 2013, by highlighting the importance of their elimination [33]. At the same time, another of the WBG’s arms – the IFC – has ramped up its health lending by investing in private health businesses as well as using capital markets since the 2000s, especially for health infrastructure, through initiatives like its – now defunct – controversial Health in Africa program [41].

Third, understanding the WBG’s influence requires careful consideration of its modes of engagement with other institutions within the global health landscape, and with the private sector, which give it a unique – and powerful – position in this landscape. The Bank’s economic power is further described in the section on the financing domain below. The rise of voluntary funding to United Nations agencies in the 1970s and 1980s [24] led to financing gaps for some core WHO and United Nations Children’s Fund (UNICEF) health programming, particularly for immunization, in the 1990s. Through its trust fund (that is, extra-budgetary aid) health portfolio, the Bank was increasingly able to capture private capital for global health in the 1990s and 2000s. This enabled it to act as financial trustee of (and sit on boards of) burgeoning partnerships with the WHO and other multilateral global public-private partnerships for health, like the Global Fund to Fight AIDS, Tuberculosis, and Malaria and Gavi, the Vaccine Alliance [11, 79]. Such a wide-reaching financial network makes the WBG an attractive lender to countries from the Global South, and accessing Bank funding has been regarded as a test of confidence, opening the door to negotiations with other donors. However, it also means that it can be hard to tease-out the Bank’s influence relative to that of other major global health institutions, such as its role in undermining United Nations agencies’ ability to fulfil their mandates. Tracing the WBG’s influential health policy knowledge is particularly complex as it is often produced in partnership with other global health institutions [23], like in the case of the introduction of the disability adjusted life year (DALY) and the healthy life expectancy (HALE) metrics, which resulted from research partnerships with the WHO and Harvard University [69]. Among other users of these metrics, the WHO and the World Bank have used the DALY and the HALE to create both their global health estimates and baselines for monitoring progress towards universal health coverage [82, 83]. Together, these forms of influence – and the challenges to capturing this influence – indicate that the Bank has the ability to develop internal health agendas, pitch them to countries using its contacts within ministries of health and ministries of finance, provide financing, obtain legitimization through other global health actors (like the private sector and civil society organizations), and ultimately influence the agendas of United Nations agencies.

## Three proposed domains for studying the World Bank’s influence on Global Health

Drawing on this broad understanding of the World Bank’s influence and challenges to capturing it, we propose three domains for studying the Bank’s influence and power in global health (Additional file 1: Table 1). In doing so, we assert that any social scientific or historical investigation must be careful not to simply amplify the organization’s own historiography and institutional ethos, as the WBG has been prolific in creating representations of its influence and work in the health sector. We highlight some of the limitations of each of these domains, and consider how a careful use of source material may help address these limitations. As discussed above, these domains are overlapping and the same forms of power may be at play across many of them – they are best seen as three categories for understanding the Bank’s discourse and actions with three different objects of study as their lenses. They represent a matrix through which researchers may study the WBG’s position in the evolving global health landscape, the ways in which the Bank’s position on different global health concepts – like universal health coverage – have changed over time, and how these changes have impacted health priorities and provision in the Global South.

### Knowledge-based: shaping health policy knowledge

The first important object of study is the knowledge that the Bank produces about health policy and project implementation. Two of Moon’s [44] forms of power are central to understanding the WBG’s role here: *expert*, or shaping “what others consider to be legitimate knowledge,” and *discursive*, or shaping “the language others use to conceptualize, frame, and thereby define and understand an issue.” The World Bank produces extensive research to help shape country-level health policy and global health initiatives through various means, including through what is now known as the Development Research Group (DRG). In its 1998 *World Development Report*, “Knowledge for Development,” then-Bank President James Wolfensohn embraced the idea of the Bank as a “Knowledge Bank,” in what Broad [9] has argued was a push to reassert the Bank’s legitimacy as its financial influence waned in the mid-1990s. The institution’s discursive and expert power can be seen in the ways that concepts and theories are put forward in the “technical” assistance the Bank provides. Such assistance takes the form of training and policy guidance, and it goes hand in hand with both direct lending or participation in global health partnerships and debates in spaces of global health governance, including working groups like the Inter-agency and Expert Group for Sustainable Development Goal Indicators (IAEG-SDGs) or events like the United Nations High-Level Meeting (UN HLM) on Universal Health Coverage.

There has been both continuity and change in the Bank’s concepts and theories that have gained purchase or have been rejected in these global governance spaces. For instance, the Bank has consistently applied theories of human capital to the health sector since the 1970s; it linked the economic “stock” of healthy individuals to disease control and nutrition during Robert McNamara’s tenure as Bank President in the 1970s [61, 78], doubled-down on human resources as a critical contributor to economic growth in the 1980s [5], and recently put forward the human capital index metric [35, 66]. Yet, its embrace of privatization has been much more punctuated. While the Bank’s 1975 *Health Policy Paper* and 1980 *World Development Report* warned against heavy emphasis on cost-benefit analysis, user fees, and private sector delivery, health economists such as David de Ferranti advocated for a more pluralist mix of public, insurance, and voluntary sector funding in the early 1980s, including user fees for public institutions [4, 67]. Although user fees have been critiqued by some within the institution [13, 33], this pluralistic approach has continued with the Bank’s advocacy of universal health coverage.

Social scientists have shown that, while the Bank has made a deep impression on global health, health economists and other experts at the Bank often work in coordination with other global health institutions or amplify longstanding health research. Since the 1980s, scholars have reflected upon the Bank’s success in infusing languages (and underlying ideologies) of economics into international and global health [3, 11, 18, 32, 56]. Others have effectively shown how many of these concepts predated World Bank involvement in global health, and that the Bank’s influence must be understood in a longer trajectory of health development [23, 73]. Some argue that there has been a shift towards economic knowledge at the expense of other forms of reasoning at the Bank, in ways that have marginalized rights-based discourse [58, 59] and particular country-specific contexts [43, 67] in its policies and program implementation. Further, Walker (2019) has highlighted that the Bank’s emphasis on economic management in health has not necessarily been matched by a consistent application of economic tools to health project planning – pointing again to the importance of studying the Bank’s shifting and heterogeneous approaches, and disconnects between discourse and practice.

This research domain sits within a larger scholarly conversation about how international organizations’ knowledge production is a key mode of producing and maintaining power in the global governance space. The Bank’s discursive and expert power in shaping economic, political, social, and environmental knowledge extends far beyond its health work, and scholars have shown how the Bank has leveraged these forms of power to set itself apart in the international development landscape. These include the Bank’s use of country-level policy and institutional benchmarks as a “governing tool” for asserting authority over both recipients and donors of Bank funds [57], and how the Bank, as a “strategic knowledge institution,” has promoted “smaller and better government” in its influential knowledge about good governance ([16]:118). Nay [46] shows how, alongside the OECD, the World Bank has played an institutional role in the “consolidation and perpetuation of the aid donors’ policy doctrine,” here, by producing knowledge about the concept of the “fragile state” and protecting the concept from “dissent” in the process.

With these cases in mind, future social scientific research into the World Bank’s role in shaping policy knowledge and expertise about health should consider two main questions. First, which actors are (and are not) involved in producing knowledge at the Bank? In other words, whose research agenda drives the Bank-initiated production of knowledge? How involved are local scholars in the production of knowledge relative to more powerful Bank staff and “clients” (like major donors), and whose interests does evidence serve? Second, how are the data so produced used? Do they guide health policy-making, or are they used post-hoc to justify decisions already made? The latter questions focus on the functional use of estimates like the global burden of disease (GBD), and may be most relevant in the case of performance- or results-based financing, as discussed further in the finance section below. This is particularly important because recent research has indicated that the metrics and tools put forward by the Bank are often not used to assess loans, due to their irrelevance to complex political negotiations [19].

We recommend that scholars undertake country or programmatic case studies for this research domain. For instance, how have in-country WBG officials leveraged global estimates of disease, or advocated for local data collection on maternal and child health? Or, how have different entities within the WBG defined the concept of “universal health coverage” [70], “right to health” [58], or “gender equality” [60]? What factors influence discourse around these concepts and what influence do they have on in-country population health and/or programmatic outcomes? Such case studies would further enable health development leaders in countries that receive Bank funding to contextualize health policy recommendations and advocate for more country-owned means of knowledge production, and to highlight the ways that contextual knowledge can be used in health development policy. These case studies may be best accomplished through discourse analysis and qualitative thematic analysis of documents, particularly of archival data at the WBG Archives, partner nation archives, and key informant interviews. In theory, the World Bank could be a strong ally in this advocacy: a strength of the Bank’s country-based model is that it is uniquely placed to promote local capacity for development, including championing the in-country production of health data over the use of estimated data [75].

### Governance-based: shaping global health governance

Our second recommended research domain is the role of the WBG since the 1980s in changing the nature of health governance at the supranational, national, and sub-national levels. Here, the two most important forms of power are *institutional*, or the “use of rules and decision-making procedures to shape decision-making,” and *network*, or the use of “personal relationships with others to shape their thinking and/or action” ([44]:6–7). The Bank is institutionally different from other global health actors like UN agencies (including the WHO), philanthropic organizations, and bilateral donors. It is an organization with member states whose votes matter more, at the end of the day, based on how much money they provide to the institution. Unlike the WHO, UNICEF, or other global health donors, its point of contact on the country level are ministers of finance, linking it more closely to those with the funds to back up its policy recommendations. With the diminishing influence of the WHO, due to the freezing of its budgets and the rise of earmarked health funding [10, 11], the Bank became one of the most important global health financiers in the 1980s, although HNP financing has certainly fluctuated in the decades since. However, like its insertion into the health policy knowledge world, officials at the WBG have introduced new ways of staking a financial claim in the global health landscape. For instance, in the push to increase investment from “billions to trillions,” the WBG is positioning itself to de-risk private equity; new financing mechanisms include trust funds to finance outbreak response by channeling funding from reinsurance and bond markets [65], impact investments for the Sustainable Development Goals (SDGs), and IFC financing for health projects, which now include specific budgetary allocations for blended finance [15].

Research is needed that situates the WBG’s influence relative to other global health actors and borrowing countries through two inter-related questions. First, to what extent does the WBG’s financial power affect its power to influence health policy at the country level? This question considers the ways that the WBG has influenced countries’ health financing. It also highlights the WBG’s relationship with ministries of finance, which is a privileged position in comparison to the WHO’s point of contact on the national level being ministries of health (e.g. [64]). Second, it is important to investigate the ways that the WBG has supported both vertical and horizontal programming – through the IDA and the IBRD’s main modalities (project financing and associated policy advice), the IFC’s investments, and the MIGA’s risk reduction for investment. That is, in what ways has the organization supported attention to single-issue health interventions or vertical approaches like interventions into HIV/AIDS or malaria, in comparison to its attention to overall health systems strengthening or horizontal approaches to health development? How has this impacted countries’ overall health system strengthening activities? To what extent has the IFC’s focus on the private sector constrained – or facilitated – the WBG’s ability to invest in primary health care?

To study these questions and situate the Bank within a complex web of global health partners, we recommend three methodological approaches. A first approach is to perform network analysis to map the connections formed by the WBG and ministries of finance and health (or other high-ranking cabinet members). This would be best accomplished by performing interview-intensive or ethnographic case studies on the country level in countries that receive Bank funding. A second country-level approach focuses on the congruence between the Bank’s formal country investment plans and the health priorities voiced by client countries. For instance, researchers could identify health priorities stated in Bank country-specific five-year action plans; analyze strategic health policy documents from the national government during this time period, which are produced with Bank and WHO staff as well as other stakeholders; and perform interviews with national stakeholders and staff from the Bank and other major global health actors to understand the programs that were ultimately implemented during this time period. Such a document and interview-based methodology would allow for a more accurate understanding of how (and whether) the Bank met country and national priorities, and what health areas were neglected.

The third approach is to investigate the Bank’s role in vertical health interventions, by studying how silos form in global health. This approach could use case studies, based on archival research and interviews at Bank headquarters, of institutions that were part of the vertical intervention, and a few countries that were beneficiaries of the intervention. It would use these case studies to analyze how funding decisions are made for particular health areas (including the evidence used, most influential voices, and decision-making structure). For example, researchers could investigate how the silo for investing in HIV/AIDS interventions developed and why no such silo has developed for intervening on mental health. Focusing on the Bank’s relative role in this prioritization process would allow for a more nuanced understanding of how the Bank has promoted (or not promoted) what it itself calls “health system strengthening.” It could also provide insight into the Bank’s influence on silo formation relative to other major global health agencies or financiers.

### Finance-based: shaping health financing mechanisms

Our final proposed research domain puts health financing at its center. The two of Moon’s [44] forms of global governance power that are most important here are *economic*, or the “use of material resources” to shape action and thought, and *institutional*, which is described in the section above. While the Bank’s ideological approach to its health financing agenda has changed since it sanctioned direct lending in 1979, it has consistently retained economic power through the sheer quantity of its lending. During the late 1970s and in its 1980 *World Development Report*, the Bank ideologically aligned with the basic needs approach, and largely supported the primary health care movement championed by the Alma Ata Declaration [67]. From the early 1980s through the 1990s, however, concerns about the affordability of universal primary health care drove the WBG and UNICEF towards selective primary health care [12], and the Bank embraced market-based resource allocation through its Structural Adjustment Programs in an attempt secure it [6, 52, 67]. As part of the Structural Adjustment Program model, the Bank embraced user fees at the point of service. The user fee debate has been covered well through case studies, which may be best summarized as pointing to their “sustainable inequity” [48], or enabling of some service provision at the deep expense of equitable access (e.g., [21]). Robert and Ridde’s (2013) documentary analysis indicates that the majority of global health actors surveyed from 2005 to 2011 favored either the abolition of user fees or free care at the point of delivery, with the Bank as the only actor that still favored both user fees and free care at the point of service.

As it prepared its health financing agenda for the new millennium, the WBG’s Operations Evaluation Department (the predecessor to the IEG) argued that, “despite an initial focus on government health services, the Bank is increasingly focusing on issues of private and nongovernmental organization (NGO) service delivery, insurance, and regulation,” and stated that clients viewed it as a powerful actor for donor coordination [31]. Indeed, since 2000, IBRD and IDA have shifted their language toward increasing public expenditure in health and attention to health systems strengthening [68], although the relative growth of the Bank’s trust fund financing for public-private partnerships compared to its core health sector lending indicates that this shift may be somewhat rhetorical [64]. The Bank’s embrace of health system strengthening and UHC have been complicated by the relative rise in power of the IFC and the WBG’s recent renewed interest in insurance as a means to secure universal health coveragee. The relative percentage of health and education within the IFC’s portfolio, for instance, rose from 2 % in 2007 to 8 % in 2015, and it has championed private health sector commitments for diagnostic chains, health insurers, information technology, and medical education [28]. In 2014, the HNP Global Practice was launched to accelerate progress towards UHC, and to build on the WBG’s 2013 Corporate Strategy of “One World Bank Group,” embracing public and private solutions. However, as pointed -out by the Independent Evaluation Group [29], collaboration between the IBRD/IDA and the IFC may be more rhetoric than reality, with a single Global Lead for Harnessing the Private Sector leading coordination activities. The contrast between the WBG’s stated support of UHC and the rise of IFC’s privatized investments in health has attracted surprisingly little scholarly attention.

How have researchers unpacked the financing mechanisms that fuel these financing ideologies? The World Bank’s unique position in the terrain of financial governance because of the size of its health lending portfolio cannot be overstated. Core World Bank funding for the health sector (HNP programs) totaled approximately $17.5 billion from 1985 to 2000, and $60.9 billion from 2000 to 2015 [78]. As Sridhar et al. [64] have documented, the theme allocation for HNP funding has changed significantly over time, with the largest portion (44%) targeted to reproductive, maternal, and child health from 1985 to 2000, and the largest portion (34%) going to budgetary support by 2000–2015. Yet, the Bank’s reported HNP data is only the “tip of the iceberg,” in terms of its involvement in financing mechanisms for projects that impact health.

At least three additional factors contribute to the WBG’s influence on health financing. First, regional and global trust funds for health activities have proliferated as financing mechanisms since the 1990s, and are largely not captured by HNP financing data. A few studies have analyzed the reasons for the proliferation of trust funds in the 1980s and 1990s, especially related to geopolitical forces at the United Nations in the 1980s and 1990s (e.g., [17, 72]). However, comprehensive thematic analyses of trust funds held at the Bank, particularly financial intermediary funds (modalities used to support Global Partnership Programs) and IFC trust funds, have not been undertaken. The IEG’s [29] report quantified health sector support to IFC and IBRD/IDA trust funds – which by 2016 supported approximately 29% of all health services projects – but did not perform a deep analysis of financial intermediary funds. These funds are typically used to support national or global public goods, often in collaboration with specialized United Nations agencies (particularly the WHO and UNICEF). The Bank is not a passive "banker" for most of these Global Partnership Programs [64]. For instance, as of 2016, the World Bank Group was represented at the governing body of twenty-two of the twenty-five existing Global Partnership Programs. This has provided the Bank with a distinct, influential network of high-level national and international stakeholders [29].

Second, the Bank also exerts economic power on the global health agenda through projects that are not technically in the health sector. As examples, many HNP projects and trust funds are targeted at humanitarian and sanitation projects in post-conflict states, and some long-standing trust funds, such as the Consultative Group on International Agricultural Research (CGIAR) and the Cities Alliance fund, support activities related to food security and slum upgrading [30]. Third, health projects can serve as a vehicle for building relationships with the Bank and "leapfrogging" into wider lending arrangements (e.g., [34]).

To further unpack the Bank’s economic power and financial influence, we identify three main research questions: (1) in what ways has the WBG influenced the way that the “public” and the “private” are conceived in global health, (2) to what extent and through what vehicles has it embraced a “selective” approach to primary health care, and (3) what types of influence has it exerted through its trust fund infrastructure, such as through performance-based financing and its financing of Global Partnership Programs?

The first question can be approached by “following the money.” How much money is allocated to states and the private sector in a country portfolio in a given number of years, based on official project and disbursal reports? The Global Health Governance Programme has analyzed Bank financing, including trust funds, by the IBRD and the IDA for health themes (e.g., [53, 68]; Winters et al. 2017 [64]), but scholars have not compared IFC, IBRD, and IDA country loans and grants in a health-specific context. This comparison of private health investments across the WBG should be investigated with special focus on any associated redistribution of social benefits, especially at the national level. Tracking country investments may require archival or document-based research and interviews in member states, as the Bank’s main modality is a government loan or grant, which may lack full transparency in disbursal reports. It can also be approached by studying the ideological underpinnings of privatization across the Bank. For instance, researchers could track the way that states and the market have been discussed by Bank employees, and how this has changed over time, through oral histories and document analysis. Has the market-orientation of the Bank’s involvement in health in the 1980s and through its 1993 *World Development Report* been replaced with other conceptualizations of health equity, and how do these new visions position health within the Bank’s larger development agenda? Finally, research on privatization should focus on the presence or absence of policy coherence between different Bank entities, especially between the IBRD, the IDA, and the IFC.

The second question builds on Structural Adjustment Programs and the Bank’s lack of buy-in to the primary health care movement. This area requires a deeper investigation of the links between UNICEF and the World Bank, which have often been closer than the Bank and the WHO (e.g., [76]). In this context, "following the money" may be complicated, due to low transparency of early trust funds at the Bank [79]. A document-based analysis of the Special Grants Program and Development Grant Facility, including financial records and memoranda from the WBG Archives, may be most effective. This research could be supplemented with oral histories and interview-based methods, with both World Bank staff and national staff involved in collaborative global programs.

For the third question, it would be productive to perform deeper analyses of the Bank’s performance- and results-based financing programs and Global Partnership Programs. Results-based financing has been used by the Bank over the last twenty years incentivize performance and improve efficiency of service delivery in LMICs [51]. The Health Results Innovation Trust Fund (HRITF), launched in 2007 and closed for fundraising in 2017, was designed to build and share an evidence base from results-based financing in the health sector, and represents the Bank’s newer model of coupling country grants with IDA lending. It has been replaced by the heavily-funded Global Financing Facility in Support of Every Woman Every Child (GFF), which incorporates a similar model of "catalytic aid" [27, 29]. To critically approach the HRITF and GFF and understand the diffusion of performance-based financing at the global level, it may be helpful to begin with critical discourse and documentary analyses. Taking Gautier and Ridde's [20] and Robert and Ridde's [55] analyses of user fee favorability and HRITF politicized evidence, respectively, as examples, sources for this research could include public documents from official websites (both the Bank and participating governments). For projects with Implementation Completion and Results (ICR) available, researchers could use Malik and Stone’s [40] coding system to further understand private actors’ potential influence over project evaluation and enforcement of conditionality.

Analyzing the complex influence that the World Bank exerts relative to other global health actors through performance-based financing and Global Partnership Programs may require deeper access to its experts. This can be provided through ethnographic approaches and oral histories, such as those conducted by Lie [39] on the World Bank-Uganda partnership. Ethnographies may be most appropriate to disentangle the Bank’s collaborations with the IFC (including at a country level), its financial relationships with ‘clients’ within country portfolios, and its influence – and role as a broker – on financial intermediary fund boards.

## Conclusions

We have argued that studying the WBG’s influence on global health requires an acknowledgement that the institution is not a monolith and that the boundaries of “health” may be blurred; that different WBG entities’ approaches to financial and technical assistance have changed in the four decades since the WBG first engaged in health development in earnest; and that the WBG often works in concert with other global health partners. It also requires an understanding that major global health actors’ self-reporting of their programmatic impacts may differ substantially from the downstream effects of these programs on local health systems or communities, a phenomenon that Rajoktia [54] has called the “success cartel.” For instance, the Bank has crafted success narratives by selectively using cost-benefit and cost-effectiveness analyses (Walker 2019 [7]; Birn 2011 [78];).

It is challenging to study a single player in the complex landscape of global health governance. This is particularly the case for the WBG, because it prefers to operate behind the scenes, has multiple arms, forms partnerships with many other players, and has projects that extend beyond the health sector. Research on the WBG is therefore vulnerable to “riding the tiger” or promoting its self-produced image, such as its strengths as a “Knowledge Bank,” as a producer of catalytic aid, and as a promoter of health system strengthening, as well as its asserted reversal from the neoliberal underpinnings of its structural adjustment policies. At the same time, the WBG is both an interesting and important object of social scientific study. The magnitude of its health lending to countries in the Global South – coupled with its unique in-country contact points, tradition of close concertation with the IMF and private capital, and deep interest in providing market-based incentives for health – give it a strategic and privileged position in global health priority setting, implementation, and impact analysis.

We have developed a research agenda to guide a more nuanced understanding of the WBG’s influence on global health since the 1970s, and the ways that its policies and investments are shaped by larger power dynamics between different bilateral and multilateral agencies and UN member states. This research agenda contains three dimensions: knowledge-based, governance-based, and finance-based, which are best viewed as overlapping lenses for analysis. We argue that multiple social scientific disciplines and methodologies together can contribute to discerning the complexities of the WBG’s influence. At the end of the day, helping countries to establish the basis for development is the main reason for which the Bank was created and is a mechanism through which it has gained legitimacy in the health sector. Our proposed research agenda will provide a step towards both holding the WBG to account for its constructs and actions, and helping it operate in ways that address the needs of the countries to which it lends.

## Supplementary Information

**Additional file 1: Table 1.** Domains for studying the World Bank’s influence in global health. Domains, objects of study, and forms of power crucial for understanding the World Bank Group’s influence on global health. (Based on models developed by Sophie Harman (see also [26]), Suerie Moon (see also [44]), and Anuj Kapilashrami, and input from other authors.)

## Data Availability

Not applicable.

## Footnotes

1 In this review, we specifically use the term ‘World Bank’ (or ‘Bank’) to refer to the International Development Association (IDA) and the International Bank for Reconstruction and Development (IBRD), or the traditional Health, Nutrition, and Population (HNP) sector. ‘World Bank Group’ (or ‘WBG’) is used when considering the Bank’s wider interaction with the International Finance Corporation (IFC), the Multilateral Investment Guarantee Agency (MIGA), and private markets.
