# Supplementary material for: Interrogating the World Bank’s role in global health knowledge production, governance, and finance
Source: Global Health. 2021 Sep 19;17:110. doi: 10.1186/s12992-021-00761-w (PMC8449994; doi:10.1186/s12992-021-00761-w)
Supplement: Supplementary file 1 — Additional file 1: Table 1. Domains for studying the World Bank’s influence in global health. Domains, objects of study, and forms of power crucial for understanding the World Bank Group’s influence on global health. (Based on models developed by Sophie Harman (see also [26]), Suerie Moon (see also [44]), and Anuj Kapilashrami, and input from other authors.) [file 12992_2021_761_MOESM1_ESM.docx]

Table 1: Domains, objects of study, and forms of power crucial for understanding the World Bank Group’s influence on global health. (Based on models developed by Sophie Harman (see also Harman 2012), Suerie Moon (see also Moon 2019), and Anuj Kapilashrami, and input from other authors.)

| **Domain and object of study** | **Most important forms of power (Moon 2019)** | **Description** | **Examples** |
| --- | --- | --- | --- |
| **Knowledge-based: shaping health policy knowledge** | Discursive, expert | Technical assistance provider, metrics producer, discursive power, strategic position (asymmetrical status of Bank and partner nations) | Dissemination, amplification, and retraction of concepts like user fees, plural forms of health cost recovery, and human capital; selective primary health care promulgation; DALYs and other morbidity and mortality estimations based on “disability” |
| **Governance-based: Shaping health governance** | Institutional,  network | Increasing work with private actors and civil society and non-governmental organizations, structural power | Promotion of and engagement with public-private partnerships (PPPs); dilution of WHO normative power through these PPPs and trust funds; financialization of health through pandemic/Ebola bonds; imposition of strict lending conditionalities |
| **Finance-based: Shaping health financing mechanisms** | Economic, institutional | Country-based lender of last resort, global lending and financing facility, economic resource provider | Creation and promotion of “innovative financing” for sustainable development goal (SDG) implementation; impact investing and de-risking private equity; pushing of concepts of catalytic aid for donor investments in trust funds |
